# Diagnosis of a severe congenital anomaly: A qualitative analysis of parental decision making and the implications for healthcare encounters

**DOI:** 10.1111/hex.12664

**Published:** 2018-02-02

**Authors:** Robyn Lotto, Lucy K. Smith, Natalie Armstrong

**Affiliations:** ^1^ Faculty of Education, Health and Community Liverpool John Moores University Liverpool UK; ^2^ Department of Health Sciences University of Leicester Leicester UK

**Keywords:** congenital anomaly, decision‐making, fetal anomaly, pregnancy, qualitative, termination of pregnancy

## Abstract

**Objective:**

To explore parental decision making following diagnosis of a severe congenital anomaly, and implications for healthcare encounters.

**Design:**

Qualitative semi‐structured interviews with 38 parents‐to‐be were collated and triangulated with data generated from consultation recordings.

**Analysis:**

Data were analysed using a constant comparative‐based approach.

**Setting:**

Recruitment was undertaken across four fetal medicine sites in two tertiary referral trusts.

**Participants:**

Parents‐to‐be whose pregnancy was suspected or diagnosed as being affected by a severe congenital anomaly. This sample was purposive to include known factors affecting the decision to terminate or continue the affected pregnancy.

**Findings:**

In trying to make a decision about how to proceed with their pregnancy, parents‐to‐be typically had to work hard to negotiate multiple uncertainties around the diagnosis and prognosis of the suspected anomaly. This was influenced by parents’ capacity to cope with uncertainty and the way in which uncertainty was managed by the clinical team. This negotiation of uncertainty was enacted within a fluid, nonlinear three‐phase process: “information seeking,” reflecting the way parents‐to‐be face the uncertainty associated with a fetal diagnosis and associated prognosis; “implications,” where consideration is given to future consequences of the decision; and “decision making,” which reflects the way in which the decision is made (head‐ or heart‐led). Spectrums of responses were apparent within each phase.

**Conclusions:**

This study provides important insights into how parents‐to‐be make decisions following diagnosis or suspicion of a severe congenital anomaly. The impact of these on healthcare encounters is discussed, alongside recommendations for clinical practice.

## 1 BACKGROUND

Most parents‐to‐be embark on a pregnancy assuming they will have a healthy child, but in around 3% of pregnancies, a lethal or life‐limiting anomaly is present.^1^ In the United Kingdom (UK), parents‐to‐be are offered antenatal screening for 11 congenital anomalies: serious cardiac, anencephaly, spina bifida, renal agenesis, lethal skeletal dysplasia, congenital diaphragmatic hernia, trisomies 13 and 18, cleft lip and gastroschisis through the Fetal Anomaly Screening Programme (FASP). The first nine of these anomalies may be defined as “severe” as they carry a significant morbidity or mortality risk, and depending on a number of factors, parents‐to‐be may be offered the option to terminate the affected pregnancy. The other anomalies, cleft lip and gastroschisis, benefit from antenatal or postnatal treatment.^2^ In England and Wales, around 70% of women terminate pregnancies affected by a FASP anomaly.^2^

Existing evidence has linked a range of variables, such as gestational age at diagnosis, severity, type of anomaly, religion and socio‐economic status, to women's decisions to continue or terminate affected pregnancies.^3^, ^4^, ^5^ However, conflicting findings demonstrate the difficulty of attempting to understand this complex decision‐making process.^5^, ^6^ For example, several papers suggest maternal age is an important influencing factor yet no consensus exists on the direction of influence; some papers conclude that younger women are more likely to terminate (and conversely older women continue),^7^, ^8^, ^9^ and another suggests the converse.^10^ Therefore, whilst these studies may provide some insight into factors that are influential, the examination of such variables is unlikely to capture effectively the complexity of the decision‐making process.

Furthermore, there is a lack of research underpinning care provision in this area, with a full understanding of what is important to parents‐to‐be as they make this complex and distressing decision, and how they go about doing so, necessary to ensure all those facing this decision are being well supported.^5^, ^9^, ^10^, ^11^ Here, we provide insights into how parents‐to‐be whose pregnancy is affected by a severe congenital anomaly make the decision about whether to continue their pregnancy or not, and the implications for healthcare encounters.

## 2 METHODOLOGY

A qualitative approach comprising triangulation of one‐to‐one narrative interviews with parents‐to‐be and audio‐recordings of clinical consultations was employed to best understand the complex reality of parental decision making.^12^ Over 80 hours of recorded data were collated. A total of 20 mothers‐to‐be and 18 partners were recruited from four tertiary referral centres (across two hospital trusts). Parents‐to‐be were initially approached by a clinician, with all those invited subsequently participating in the study. Inclusion/exclusion criteria were broad to include any pregnancy affected by a severe anomaly, as defined by FASP. Interviews were undertaken with mothers‐to‐be, and their partners together where possible (n = 15), with five mothers‐to‐be interviewed alone. The remaining three partners contributed to the data collated during the consultations. Sampling was purposive to ensure a range of “severe” diagnoses where termination would be an option offered, gestational age at diagnosis, ethnicity, socio‐economic status and, ultimately, the decision made of whether to continue or terminate the affected pregnancy. Patient and public involvement was an integral part of all aspects of the research, with specific advice sought on the feasibility and acceptability of proposed recruitment timing and strategies.^13^ Interviews and consultations were audio‐recorded with consent. Data generated were anonymized and transcribed verbatim. Analysis was undertaken using a constant comparative‐based approach.^14^ Methods of comparison were formalized in a three‐step process. First, each item of data, whether interview or consultation, was coded and internal were comparisons made to highlight difficulties or inconsistencies. Second, comparisons were made across data pertaining to the same participant, thus comparing data derived from consultations and interviews. Third, the process was repeated across the couples.

Ethical permission was granted by the Nottingham Research Ethics Committee (REC reference 13/EM/0293).

## 3 RESULTS

### 3.1 Parental decision‐making themes

The decision‐making process enacted by parents‐to‐be is complex and individual. However, a consistent over‐arching theme of “negotiating uncertainty” was identified. This relates to the way in which parents‐to‐be navigate and negotiate the intrinsic uncertainty arising from a diagnosis of a severe congenital anomaly. The elements underpinning this theme included the following: the level of uncertainty created by the anomaly detected; capacity of parents‐to‐be to cope with uncertainty; and the way in which uncertainty was managed by the clinical team. “Negotiating uncertainty” is enacted within a fluid, nonlinear three‐phase framework: information‐seeking, reflecting the way parents‐to‐be face the uncertainty associated with a fetal diagnosis and associated prognosis; implications, where consideration is given to future consequences of the decision; and decision making, which reflects the way in which the decision is made (head‐ or heart‐ led). Spectrums of responses were apparent within each phase.

### 3.2 Information‐seeking

Parents‐to‐be encountered difficulties in coming to terms with the diagnosis and prognosis of the congenital anomaly. The complexities of the diagnostic process, as well as their degree of understanding, influenced the ability of parents‐to‐be to alleviate their uncertainty surrounding the diagnosis.

Visibility of the anomaly contributed significantly to parental acceptance of the diagnosis. In cases where the absence of all or part of an essential organ was visible to the parents‐to‐be on the scan, acceptance of the diagnosis was easier.

We could see the big hole [in the head]; I mean it was obvious really. He was never going to survive. (Mother06–Terminated)

However, this was not the case for all structural anomalies. Where clinicians were required to interpret the tests (in the case below, an ultrasound scan), parents’ acceptance was variable and reliant on them having trust in the clinician.

I've no idea how they see anything on those things [scans]. I mean I guess we just have to trust them [the clinicians] (Mother02‐Terminated)

Unlike scans that can be interpreted by the operator at the time of the procedure, invasive testing such as amniocentesis or chorionic villus sampling (CVS), where a sample is sent to the laboratory, requires time to culture and analyse. The impact of this waiting time is significant. Tests such as the fluorescence in situ hybridizsation (FISH) test have been utilized to provide quick (24 hour) results. For many parents‐to‐be, this provides a rapid end to uncertainty through a reliable test with a zero false ‐positive rate. However, in the event that a mosaicism (an incomplete chromosomal anomaly, where not all the cells are affected) is identified, the FISH test may present a false‐negative result, resulting in further uncertainty for the parents‐to‐be. As one father explained:

The results from the very first tests came back normal, so we thought there wasn't a risk. So it wasn't until [a few weeks later] that we were told that the full culture had come back positive [to a mosaic chromosomal anomaly], I mean what are you supposed to think? (Father07–Continued–Consultation4)

In this instance, the parents‐to‐be had received negative results from the FISH tests. The positive full culture results were then perceived as questionable. Lack of understanding or tolerance of the uncertainty resulting from these conflicting results appeared to prevent these parents‐to‐be fully accepting the diagnosis or prognosis.

One strategy for alleviating uncertainty and reaching acceptance widely discussed by the parents‐to‐be was information‐seeking. This manifested in two ways: activities such as Internet searches or accessing organizations online; and participating in monitoring or testing to obtain additional clinical information or provide confirmation of the potential diagnoses or prognoses. Parental approach and attitudes in both cases varied significantly. For some, validation of the information provided by clinicians was seen as an essential step towards accepting the diagnosis or prognosis, and being able to move towards making their decision, particularly if this was likely to be to terminate the pregnancy.

We organised between them telling us what it was and going in and we went on the internet… I mean we just wanted to know for ourselves that it wasn't going to… she wasn't going to live. (Father10–Terminated)

Alternatively, others responded to the identification of a potential anomaly by avoiding all information.

I don't want to read anything on the internet; I don't want to go on any websites I don't want to look at any pictures. (Mother01–Continued)

Although parents seeking information generally understood the importance of assessing the source of the information, some parents‐to‐be appeared to find more difficulty than others in identifying reliable sites.

…they had given me a list of it could possibly be this… you go home and you type in google and you click on the first link and the first link is never the proper one to look at. You need to go to google scholar or something like that to get the real ones, but you just do, don't you? (Mother10–Terminated)

For these parents‐to‐be, the variability of the information identified created the need for ongoing searches to validate their understanding. This then risked becoming a vicious circle. An assumption is often made that more information will lead to a more informed decision. However, this assumption fails to account for the complexities of the decision‐making processes enacted by the parents‐to‐be, or the influence of health literacy skills in their ability to identify, process and utilize information available.

Participation in monitoring or further testing provided additional opportunities to alleviate further uncertainty. However, invasive testing procedures such as amniocentesis or CVS carry a risk of miscarriage, thus requiring parents‐to‐be to balance the risk to their baby with the need for information to inform their decision. Here, a spectrum from unquestioning acceptance “because the doctor said so” to informed decision making and subsequent acceptance or refusal of the recommended tests was apparent.

So it just felt like for a 1% risk, which is virtually zero. It was worth it to find out. (Father02–Terminated)

Although none of the women ultimately refused further testing, some chose to delay this until the end of the pregnancy.

The decision to refuse invasive testing at this point suggests that confirmation of a severe anomaly would not influence the decision to continue the pregnancy. The acceptance of later invasive testing when the risk of miscarriage was no longer relevant reinforces that rejection of invasive testing was not an avoidance of information, but an active decision to avoid the associated risk of miscarriage.

### 3.3 Implications

Another phase of the decision‐making process related to the consideration parents‐to‐be gave to the future consequences. This involved weighing up options of varying uncertainty, primarily in relation to prognosis. Parents‐to‐be placed differing emphasis on the importance of evaluating the future impact of their choice as part of their decision‐making process. Whilst some placed a high level of importance on evaluating the potential long‐ and short‐term consequences of their actions, not only for the baby but also for themselves and family, other parents‐to‐be were either unable or unwilling to look further than the immediate situation.

The short‐term issues raised frequently related to the potential suffering of the baby.

It was the thought that the baby would suffer and be in pain… (Mother02–Terminated)

In the long term, the emphasis shifted to consideration of the impact on themselves and other family members.

But we have got to think about the other children… (Mother09–Terminated–Consultation2)

Conversely, some parents‐to‐be did not feel that the future was theirs to decide or found they were unable to separate themselves from the emotional aspect of the decision in order to look ahead.

I was, not in denial, but didn't want to think of the end circumstances. (Mother13‐Continued)

Whilst all the parents‐to‐be discussed the future impact of their decision, some were characterized by the significant emphasis they placed on their perceived need to evaluate this aspect as part of their decision‐making process. Conversely, others took a more passive stance, often unable or unwilling to consider future consequences.

### 3.4 Decision making

The third phase encompasses the way in which a decision was made, with a dichotomy in responses noted. On one side, parents‐to‐be suggested an analytical, methodical process to decision making, whilst on the other, the decision‐making process appeared haphazard, disorganized and unsystematic. For some, this risked no decision being made, and the pregnancy continuing by default. More colloquially, the differences in the processes employed could be compared to “head‐” or “heart‐” led decision‐making styles.^15^ The “head‐led” approach supported a rational/practical approach to weighing one option over the other. Conversely, the “heart‐led” approach was, as it suggests, often emotion led. Practicalities of the decision were overlooked in favour of feelings or “gut reactions” about what was the right thing to do. The excerpt below illustrates the “head‐led” approach where a practical and balanced approach to the decision‐making process is demonstrated.

We did look at the practical, we looked at what [anomaly] was and the difficulties if he did survive, and the quality of life…, we took all sorts of things into consideration really, but looked at it from a practical position. (Mother09–Terminated)

Other parents‐to‐be found it difficult to overcome the emotiveness of the situation, and appeared more “heart‐led.” The parents‐to‐be identified that they were already grieving for their lost dream of a ‘perfect baby’, and were unable to engage in the weighing up of options.

…we kept swinging from one side to the other… there wasn't any sort of real process to deciding what to do, weighing everything up was just so difficult because we were grieving (Mother15–Terminated)

No clear division between the way in which the decision was made and the final outcome (continuation or termination) was evident. However, evidence suggests that clinicians seek an idealised, “rational” decision‐making process to be satisfied a “good” decision has been made, with the ability to weigh up options an essential aspect of this process.^16^ For parents‐to‐be who were unready or unable to engage in this way, tensions between themselves and the clinicians could arise. This was particularly evident when parents‐to‐be continued the affected pregnancy; they were frustrated when clinicians seemed unwilling to accept their decision.

…every time we come in it's: “do you want this test, do you want this test, do you want this test.” We've said no. We've said NO. But they keep on, and every time they say it over and over again. (Father08–Continued)

Whilst invasive testing could offer additional information, for some parents‐to‐be, the risks associated outweighed the benefits. Repeated offers of testing were sometimes perceived as a failure on the part of clinicians to accept their perspective^17^ and were suggestive of differing attitudes to risk and uncertainty between clinicians and the parents‐to‐be.

For a small group of parents‐to‐be, no decision was required due to fundamental beliefs that termination was wrong under any circumstances (often, but not always, religious in nature).

I could see how that would have been, not the easy way out, ‘cause I don't think that would have been easy, but I could see how that would have been the ‘best’ thing for some people… But that just wasn't an option for us. (Mother01‐Continued)

Most parents‐to‐be who expressed such beliefs continued with their pregnancy, but there were exceptions.

I've always been brought up believing termination is wrong, but with all the things wrong with her, I just couldn't put her through [being born]. I mean the suffering would have been terrible. (Mother06–Terminated)

Despite being brought up with strong beliefs, these parents‐to‐be found the reality of the situation overwhelming. Their subsequent decision to terminate the pregnancy resulted in the parents‐to‐be feeling isolated and cut off from potential support networks.

I haven't been back to church since… You get all these things drummed into your head then you cannot get them out… I've been too afraid to go back (Mother19–Terminated)

## 4 DISCUSSION

The complexity of the situation faced by parents‐to‐be as they negotiate the uncertainty arising from the diagnosis of a severe fetal anomaly is increasingly documented within the antenatal literature.^16^, ^18^, ^19^, ^20^ However, the decision‐making processes and the impact of these on healthcare encounters are largely overlooked, with the findings of this study providing initial insights that begin to address this gap.

The over‐riding theme of “negotiating uncertainty” is enacted within a fluid, nonlinear three‐phase framework: “information‐seeking,” reflecting the degree to which parents‐to‐be accept or deny the uncertainty associated with the potential diagnosis and prognosis; “implications,” where consideration is given to future consequences of the decision; and “decision making,” which reflects the way in which the decision is made (head‐ or heart‐led). However, each phase is not isolated, and the process is dynamic and interactive. The spectrum of responses highlights the diversity of needs and subsequent difficulties clinicians face ensuring the appropriate and desired level of support is provided to individual parents.

The nature of the anomaly creates varying levels of uncertainty amongst parents‐to‐be.^3^, ^4^, ^5^, ^21^ The diagnostic techniques employed, and the subsequent diagnosis and prognosis, can compound the uncertainty created. Visualizing the anomaly appeared to assist parents‐to‐be to reach a level of “information‐seeking” that subsequently enabled them to move forward to engage in active decision making, or postnatally to provide reassurance that they had made the “right” decision.^22^ Conversely, where visualization was not possible, parents‐to‐be were reliant on clinicians to interpret complex scans or invasive diagnostic tests. This required a high level of trust in the competence of clinicians^23^ in order for parents‐to‐be to achieve a level of “information‐seeking” sufficient to support informed decision making.^24^ Trust in the doctor‐patient relationship can be fragile,^25^ requiring elements of both technical and interpersonal skills.^23^ It takes time to develop, but is quickly destroyed should shared understandings be lost.^25^ Within the antenatal setting, this is particularly challenging, both due to the variety of ways that an anomaly may present^26^ and the natural time limitations imposed by pregnancy gestation.^16^

“Information seeking” was a prominent mechanism employed by parents‐to‐be to overcome uncertainty. Whilst provision of additional information is frequently advocated as a solution to support complex decision making, the difficulties associated with this have also been highlighted.^18^, ^27^ These findings further support the need for a more nuanced view of informing parents‐to‐be due to the complexity and variability of the information‐seeking process enacted. In this study, the influence of the Internet and technology‐based health literacy skills were key to many parents‐to‐be. Despite acknowledging the importance of accessing reliable sources, many parents‐to‐be suggested that the emotional work required to make “rational” or “head‐led” assessment of the information could become too great, even when they possessed the skills required. This potentially suggests a role for clinicians in providing support for online searching, interpretation and application, rather than additional written information.^28^, ^29^ However, the acceptance of this form of support, where information is used as a tool to validate or “test” the clinician, remains to be explored.

Literature examining risk and uncertainty suggests that when confronted with new, overwhelming information, people often develop “blind spots” for poor outcomes.^30^ For example, a high risk of mortality may be equated to a low risk of survival, but a chance, nonetheless, with a subsequent “normal life” often assumed.^30^ This assumption has been played out publicly in the recent Charlie Gard case, where disagreement between parents and clinicians in the decision to withdraw the active treatment of an infant with a critical, life‐limiting condition was played out through the courts.^31^ Similarly, as this study highlights, differing perceptions of risk between parent‐to‐be and clinician may result in a perceived lack of support for the parents‐to‐be and result in conflict between the two parties. This suggests that there is a need to rethink the way in which outcomes are represented^32^ and further supports a change in the role of clinicians from information sources to navigators in data interpretation.

An underlying assumption of many health behaviour theories is that health decisions involve a period of deliberation by the decision‐maker, at which point the consequences or implications of the decision are weighed up.^33^, ^34^ The findings from this study suggest that this was not always the case. This is observed not only in the way the decision to engage in invasive testing was made but also in subsequent actions where the implications of the decision outcome were evaluated actively or passively. Termination is a highly emotive area, associated with intense public, media and legal scrutiny. Where parents‐to‐be do not demonstrate participation in some form of weighing up of the implications, clinician expectations and parental actions will diverge, with the potential for the clinician‐parent relationship to breakdown. Whilst continuing with an affected pregnancy may be an active decision on the part of the parents‐to‐be, it may also be a passive reaction to an uncertain situation, whereby no active decision is made and the pregnancy continues by default. Conversely, the same conditions cannot be applied to the decision to terminate an affected pregnancy, where an active decision is required, with clinicians acting as gatekeepers to access. Where a functioning clinician‐parent relationship exists, options remain available to parents. However, where expectations diverge, this may not be the case. Whilst these findings provide some insight into the complexities of the parental decision‐making process, greater awareness of the impact of the decision‐making processes enacted by parents is required.

### 4.1 Limitations

From a practical perspective, there are a number of limitations to this study that must be acknowledged. Recruitment was reliant on clinicians identifying eligible parents‐to‐be and allowing us to approach them. Although no parent‐to‐be who was invited to participate declined, we cannot be sure how many parents‐to‐be were not identified to us by clinicians and hence not invited to participate.

We excluded women who did not speak sufficient English to consent and subsequently participate. These women were likely to belong to minority ethnic groups, risking under‐representation of these groups within the final sample. The difficulties in recruiting minority ethnic participants into research are well documented,^35^ as is the importance of recruitment in order to ensure applicability of findings.^36^ Both ethnicity and religion have been identified as predictor variables for decision making following diagnosis of a severe congenital anomaly^7^, ^37^, ^38^, ^39^ and are likely reflectors of unexplored cultural and contextual characteristics.^40^ Exclusion of this group may have precluded the opportunity to identify specific or differing needs, and further research is needed in this area. Despite these limitations, this study provides the first insight into the decision‐making process of women and their partners affected by a severe congenital anomaly.

### 4.2 Implications and recommendations

The themes identified in this study provide a framework through which the decision‐making process of parents‐to‐be can be better understood and needs and expectations anticipated. Strategies to improve communication and understanding of congenital anomalies at individual and societal levels are required.

Greater public awareness of congenital anomalies and the decisions faced by parents‐to‐be is required, both to reduce the stigma associated with termination and to ensure parents‐to‐be facing these terrible dilemmas arrive at diagnosis already partially informed. Greater understanding of the ways in which uncertainty is managed and understood by parents‐to‐be is required to mitigate potential confusion in the event of a false positive or negative screening. Furthermore, research is required to identify effective ways of presenting outcomes to parents‐to‐be that is more easily accessible. In addition, the role of clinicians as navigators in data interpretation, rather than information sources per se, requires exploration. This is demonstrated in the need of parents‐to‐be to reconcile the role of the clinician as information giver, and the need to validate information independently.

Tensions between clinician and parent‐to‐be, where repeated offers of testing or treatment were made, are apparent in a number of transcripts. Identifying mechanisms for managing conflicting agendas in such scenarios is essential to avoid disengagement and loss of treatment options open to those involved. Future research leading to the development of guidance for clinicians on strategies to employ in similar scenarios would be beneficial.

Finally, the implications of the loss of social support for individuals whose decisions deviate from social or cultural norms require attention. Whilst the long‐term psychological impact of the parental decision‐making process has not been evaluated in this study, the narratives of parents‐to‐be who went against the views of their families and support networks highlight the increased psychological risk to those individuals. The development of tools for clinicians to assess the psychological risk to parents following loss of an infant would allow targeted prescription of additional support.

## 5 CONCLUSIONS

This study provides a first insight into the nature of parental decision making following diagnosis or suspicion of a severe congenital anomaly. Despite similarities with screening decisions, the two should not be assumed to be representative of the other. An evidence base specific to the decision‐making process following diagnosis of a severe congenital anomaly is required if we are to identify areas where inequalities in practice or choice exist.

## CONFLICT OF INTEREST

The authors declare that there is no conflict of interest.
